# Trajectories of 24-h movement guidelines from middle adolescence to adulthood on depression and suicidal ideation: a 22-year follow-up study

**DOI:** 10.1186/s12966-022-01367-0

**Published:** 2022-10-23

**Authors:** Antonio García-Hermoso, Yasmin Ezzatvar, Robinson Ramírez-Vélez, José Francisco López-Gil, Mikel Izquierdo

**Affiliations:** 1 Navarrabiomed, Hospital Universitario de Navarra (HUN), Universidad Pública de Navarra (UPNA), IdiSNA, Pamplona Spain; 2 Department of Nursing, Universitat de València, Valencia, Spain; 3 Department of Health Sciences, Public University of Navarra, CIBER of Frailty and Healthy Aging (CIBERFES), Instituto de Salud Carlos III, Pamplona, Navarra Spain; 4 Health and Social Research Center, Universidad de Castilla-La Mancha, Cuenca, Spain

**Keywords:** Physical activity, Screen time, Sleep duration, Mental health

## Abstract

**Background:**

The 24-h movement guidelines for youth and adults recommend the specific duration of physical activity, sedentary time, and sleep duration to ensure optimal health, but little is known about its relationship to mental health indicators. The aim of the study was to explore the association between 24-h movement guidelines in adolescence and its trajectories from middle adolescence (12–17 years old) to adulthood (33–39 years old) with depression and suicidal ideation in adulthood.

**Methods:**

This prospective cohort study included individuals who participated in Waves I (1994–1995) and V (2016–2018) of the National Longitudinal Study of Adolescent Health (Add Health) in the United States. Physical activity, screen time and sleep duration were measured using questionnaires. Adults were categorized as having depression if they had a self-reported history of depression and/or prescription medication-use for depression in the previous four weeks. Suicidal ideation was assessed by a self-reported single question in both waves. Poisson regression analyses were used to estimate the incidence rate ratio (IRR) of depression and suicidal ideation at adulthood, according to meeting specific and combinations of 24-h movement guidelines at Wave I and its trajectories from adolescence to adulthood.

**Results:**

The study included 7,069 individuals (56.8% women). Adolescents who met physical activity guidelines and all three guidelines at middle adolescence had lower risk of depression (IRR = 0.84, 95%CI 0.72 to 0.98) and suicidal ideation (IRR = 0.74, 95%CI 0.55 to 0.99) at adulthood than those who did not meet any of these guidelines, respectively. Individuals who met the guidelines for screen time and all three guidelines in both adolescence and adulthood had lower risk of depression (screen time, IRR = 0.87, 95% CI 0.72 to 0.98; all three, IRR = 0.3*7*, 95% CI 0.15 to 0.92) and suicidal ideation (screen time, IRR = 0.74, 95% CI 0.51 to 0.97; all three, IRR = 0.12, 95% CI 0.06 to 0.33) than those who never met the guidelines. Additionally, individuals who did not meet all three guidelines in adolescence but met the guidelines in adulthood had lower risk of suicidal ideation than those who never met the guidelines (IRR = 0.81, 95%CI 0.45 to 0.89).

**Conclusion:**

Our findings highlight the importance of promoting and maintaining adherence to the 24-h movement guidelines from middle adolescence to adulthood to prevent mental health problems. However, our findings must be interpreted carefully due to declared limitations, e.g., the self-reported assessments which are subject to sources of error and bias or that the dataset used to gauge meeting a guidelines (1994–1996) was made later (2016).

**Supplementary Information:**

The online version contains supplementary material available at 10.1186/s12966-022-01367-0.

## Background

Mental health disorders are a public health concern due to their heavy individual, social, and economic burdens [1]. These disorders include depression, which is the second leading cause of years lived with disability and a leading cause of disability-adjusted life years according to The Global Burden of Diseases, Injuries, and Risk Factors Study 2019 [2]. Depression is also one of the most common major psychiatric disorders that frequently begins during adolescence [3], and its negative impacts can extend into adulthood [4]. Suicidal ideation, despite not being considered a mental disorder, is also a serious global public health problem and seems to be related to these factors [5]. Globally, 703,000 people die by suicide every year according to the World Health Organization, and it is the second leading cause of death among 15–29-year-olds. Among the United States (U.S.) population, the global age-standardized suicide rate was 14.5 per 100,000 people in 2019 [6]. Therefore, it is important to identify modifiable factors that could prevent or alleviate depressive symptoms and suicidal ideation.

On the other hand, inadequate movement behaviors (i.e., physical inactivity, excessive screen time, and short sleep duration) have been previously identified as factors associated with depression[7, 8] and suicidality[9–11] among adolescents. However, these studies considered movement behaviors as independent risk factors rather than mutually exclusive parts of the 24-h continuum that affect health synergistically [12, 13]. Therefore, these three behaviors are codependent and should be considered simultaneously [14]. For this reason, the Canadian Society for Exercise Physiology convened a Consensus Panel including representatives of national organizations, content experts, methodologists, stakeholders, and end-users who followed rigorous and transparent guideline development procedures to create the Canadian 24-h movement guidelines for children and youth [15], and the Canadian 24-h movement guidelines for adults [16], which have been extensively used worldwide [17]. According to these guidelines, within a 24-h period, children and adolescents should accumulate at least 60 min per day of moderate-to-vigorous intensity leisure physical activity, ≤ 2 h/day of recreational screen time, and 9–11 h of sleep per day (5–13 years old) or 8–10 h of sleep per day (14–17 years old). Adults should accumulate at least 150 min per week of moderate-to-vigorous intensity physical activity, ≤ 3 h/day of recreational screen time, and 7–9 h per day of sleep.

Previous studies looking at the 24-h movement guidelines and health indicators have primarily focused on physical health outcomes [12, 18]. However, little is known about its relationship to mental health indicators. Two representative studies of U.S. children and adolescents indicated that meeting all three 24-h movement guidelines was associated with lower odds for depression among adolescents [19, 20]. This finding was also reported by a previous systematic review that also indicated favorable associations between meeting all three 24-h movement guidelines and lower depressive symptoms and other mental health indicators (e.g., anxiety, psychological distress, suicidal behavior, flourishing, prosocial behavior) among children and adolescents in comparison with those meeting none of the guidelines [21]. However, this review suggests that the included studies are limited either by their cross-sectional designs and very low quality [21]. Accordingly, regarding suicidal ideation, only one cross-sectional study has analyzed this association and suggests that meeting 24-h movement guidelines among Canadian adolescents is related to lower odds of suicidal ideation in older boys aged 15–20 years [22].

To date, only three studies have examined prospective associations between movement behaviors and depressive symptoms among adolescents but analyzed a short follow-up of one year [23–25]. Similarly, a prospective cohort of 1974 Chinese children aged 7–9 years provided convincing epidemiological evidence that an unhealthy lifestyle during childhood (screen time, physical activity, sleep duration, and beverage intake) was associated with a more than twofold elevated risk for suicidal ideation, nonsuicidal self-harm, and depressive symptoms over a period of 5 years [26]. Notwithstanding, no prospective study has analyzed suicidal ideation and its relationship with 24-h movement guidelines. Studies examining the prospective relationships between 24-h movement guidelines and mental health indicators from adolescence to adulthood are thus warranted. Therefore, the aims of the study were as follows: (i) to describe changes in participants’ 24-h movement guidelines from adolescence to adulthood; (ii) to explore the association of movement behavior in adolescence with depressive symptoms and suicidal ideation in adulthood; and (iii) to explore the association of changes in movement behavior from adolescence to adulthood with depressive symptoms and suicidal ideation in adulthood.

## Methods

### Population sample and study design

This is a longitudinal study with data from the Add Health study, a nationally representative sample of adolescents in grades 7–12 in the U.S. followed from adolescence through adulthood. During 1994 and 1995, over 90,000 students from a sample of 80 high schools and 52 middle were selected with unequal probability of selection completed in-school questionnaires, and 20,745 of them were selected to participate in the Wave I in-home interview in 1994 [27]. Wave V in-home sample was followed in 2016–2018 (Wave V, n = 12,300, age range 33–39 years). Those participants with missing data at baseline and at Wave V on 24-h movement behaviors (n = 3,534), and/or older than 18 years old (n = 5,286), and/or diagnosed with depression at Wave I previous than 18 years old (“*How old were you when you were diagnosed by a doctor, nurse or other health care provider with depression*?”) (n = 565), and/or suicidal attempt at Wave I (“*During the past 12 months, how many times did you actually attempt suicide?”*) (n = 471), were also excluded. The final sample included 7,069 participants (56.8% women).

A chi-square analysis was conducted to compare adolescents included in the final sample with the remaining participants who were not included in the analysis. There were no differences in both depression (p = 0.910) and suicidal ideation (p = 0.811) at Wave V between adolescents who were included or not in the final sample. Therefore, it could be assumed that missing data did not meaningfully influence results within the analytic sample.

Add Health study was approved by the Institutional Review Board (IRB) at the University of North Carolina at Chapel Hill. The permission to conduct secondary analyses was obtained by the Ethics Committee of the University Hospital of Navarra (PI_2020/143).

### Depression

Adults were categorized as having depression if they had self-reported history of depression (“*Has a doctor, nurse, or other health care provider ever told you that you have or had depression*?”) and/or any prescription medication-use for depression in the past 4 weeks. Also, adults at Wave V were asked in the prior seven days how often experienced the following symptoms: “felt depressed”, “felt sad”, “felt happy” (reverse coded), “could not shake off the blues”, and “felt that life was not worth living”. Responses to each item ranging from 0 (never or rarely) to 3 (most of the time or all the time) [28]. Higher scores indicated greater symptoms of depression (a score ranging from 0 to 15). The 5-item depression scale (5-item Center for Epidemiologic Studies Depression Scale, CES-D) was internally consistent (Cronbach α = 0.78) [28].

### Suicidal ideation

Suicidal ideation was assessed by a single question with a dichotomic yes/no response option: “*During the past 12 months, did you ever seriously think about committing suicide?*” Suicidal attempts were assessed by a single item: “*During the past 12 months, how many times did you attempt suicide?*”. This question included the following response options: “zero”, “one”, “two”, “three”, and “four or more”.

### 24-h movement guidelines

#### Physical activity

In the Wave I in-home interview, adolescents reported their engagement in moderate-to-vigorous physical activity (MVPA) during the past seven days by a previously described scale and three different questions [29]: “*During the past week, how many times did you go rollerblading, roller-skating, skateboarding, or bicycling?*”; “*During the past week, how many times did you play an active sport, such as baseball, softball, basketball, soccer, swimming, or football?*”; “*During the past week, how many times did you exercise, such as jogging, walking, karate, jumping rope, gymnastics or dancing?*”. Responses ranged from not at all to five or more times and were scored as: 0 times = not at all, 1.5 times = 1 or 2 times, 3.5 times = 3 or 4 times, and 6 times = 5 or more times. Responses to the three questions were summed to create a measure of total times of MVPA each week, classified as no (0 times), some (1–4 times), and high (5 or more times) MVPA per week. Meeting physical activity guideline was considered when adolescents reported 5 or more times MVPA per week following the Gordon-Larsen et al. criterion [29].

At Wave V, individuals reported the frequency of physical activities by answering the following questions: *“In the past 7 days, how many times did you bicycle, skateboard, dance, hike, hunt, or do yard work?”, “In the past 7 days, how many times did you roller blade, roller skate, downhill ski, snowboard, play racquet sports, or do aerobics?”, “In the past 7 days, how many times did you participate in gymnastics, weight lifting, or strength training?”, “In the past 7 days, how many times did you participate in individual sports such as running, wrestling, swimming, cross-country skiing, cycle racing, martial arts, or in strenuous team sports such as football, soccer, basketball, lacrosse, rugby, field hockey, or ice hockey?”*, and *“In the past 7 days, how many times did you play golf, go fishing or bowling, or play softball or baseball?”.* To avoid reporting of erroneously inflated physical activity levels in adults resulting from the additional items, a scaled sum of MVPA sessions was created. The total sum of MVPA bouts reported in Wave V was scaled to be equivalent to that of Wave I (by dividing by the total number of activities in Wave V [n = 34] and then multiplying by the total number of activities in Wave I [n = 16]) [29].

#### Screen time

Screen time was measured by a previously described scale [29], using the following questions: “*How many hours a week do you watch television?*”, “*How many hours a week do you watch videos?*”, and “*How many hours a week do you play video or computer games?*” The amount of hours given in the three responses were summed to create a measure of recreational screen time per week. At Wave I, meeting screen time guidelines was considered when adolescents reported ≤2 h per day. At Wave V, meeting screen time guidelines was considered when adults reported ≤3 h per day [16].

#### Sleep time

Adolescents and adults reported their sleep duration (in hours) in response to one question in the Wave I and V in-home interview: “How many hours of sleep do you usually get per day and/or night?” The prevalence of meeting sleep duration guidelines was estimated by the National Sleep Foundation’s sleep duration guidelines from 9 to 11 h and 8 to 10 h per day of sleep in those aged 12–13 and 14–17 years old, respectively [30]. At Wave V, meeting sleep duration guidelines was considered when adults reported from 7 to 9 h per day of sleep [16].

#### Trajectories

For each individual, change for each movement guidelines component was calculated as follows: (a) always met, which consisted of individuals who met the guideline at adolescence and adulthood; (b) increased, which consisted of participants who did not meet the guideline at adolescence but at adulthood; (c) decreased, which consisted of participants who met the guideline at adolescence but not at adulthood; and (d) never met, which consisted of individuals who did not meet the guideline at adolescence and adulthood.

#### Covariates

Information on sociodemographic factors, such as age, sex, race/ethnicity (operationalized as a four-level: White, Black, Native American, and Asian), and parental income (range $0 to $999 thousand), was collected through in-home questionnaires. Alcohol consumption was also assessed through the responses to the following question: “*Within the last 30 days, on how many days did you have alcohol (beer, wine, liquor)?*” participants who reported “never used” were classified as “never drinker”, those who reported “have used, but not in last 30 days” were classified as “former drinker”, and those who chose other options were classified as “current drinker”.

### Statistical analysis

Descriptive information is shown as numbers and percentages for categorical variables and mean and standard deviation for continuous variables. Preliminary analyses showed no significant interactions between sex and all three movement behaviors in relation to depression symptoms (*p* = 0.741) and suicidal ideation (p = 0.624) at Wave V; therefore, all analyses were performed with men and women together. All model assumptions were checked (i.e., normality and homoscedasticity).

**Fig. 1:**
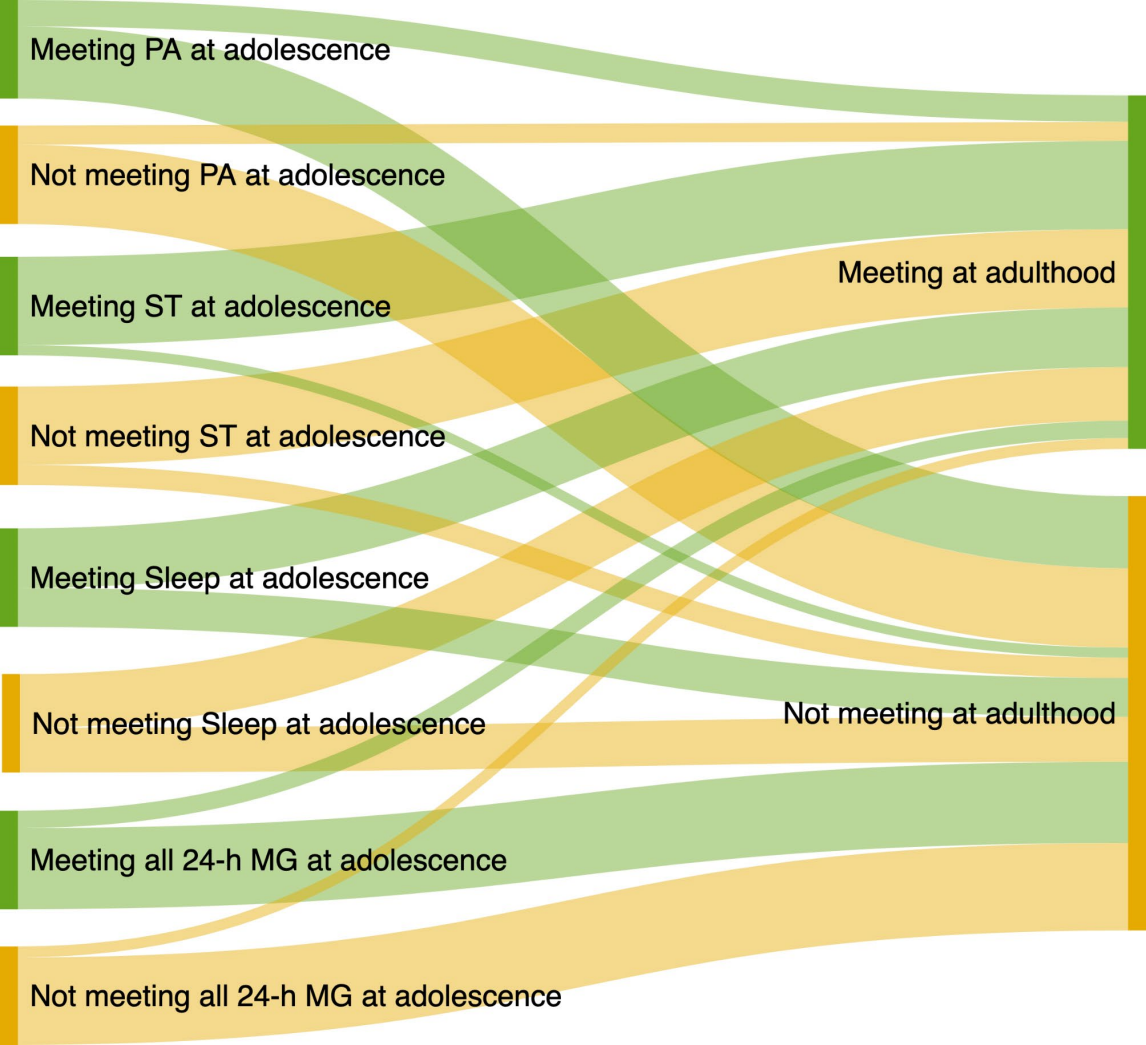
Trajectories of 24-h movement guidelines from adolescence to adulthood

To show the trajectories of 24-h movement guidelines from adolescence to adulthood based on its proportional frequency, we created a Sankey diagram (Fig. 1) by using the online tool SankeyMATIC (www.sankeymatic.com).

Poisson regression with robust error variance analyses [31] were used to estimate the incidence rate ratio (IRR) of depression and suicidal ideation (dependent variables), according to meeting specific (i.e., physical activity only, screen time only, sleep only) and combinations (i.e., all three guidelines) of 24-h movement guidelines at Wave I (independent variables). In all cases non-meeting the guideline(s) was the reference group. Also, we estimated the IRR of depression and suicidal ideation according to trajectories of 24-h movement guidelines from adolescence to adulthood using “never met” as reference group. All analyses were adjusted by sex, race/ethnicity, parental income at Wave I, age at Wave V, depression score (5-item depression scale) at Wave V, and alcohol consumption at Wave V. Further, we used mutual adjustments between physical activity, screen time, and sleep duration.

In order to correct for design effects and unequal probability of selection, we used a statistical package that adjusts for the survey design. We used STATA version 17.0 (StataCorp LLC, College Station, TX) with *SVY* commands to conduct design-based analyses that accounted for stratification, clustering, and unequal probability of selection [32] and set significance at *p* < 0.05. STATA weights the data so that it is representative of the actual composition of each school based on grade level and sex and corrects for the unequal probability of selection of schools across regions. Finally, STATA adjusts standard errors proportional to the degree of nesting.

## Results

Table 1 shows descriptive statistics for the full sample at Wave I and at Wave V. There were 7,069 respondents who participated in both waves of data collection (56.8% women, mean age at Wave I 15.35 years and 37.25 at Wave V, 71.6% White, and 38.3% obtained college degree or higher). At Wave V, 21.0% of the participants were diagnosed with depression, 332 (5.2%) adults thought about suicide, and 95 (1.3%) reported suicide attempts.

**Table 1:** Descriptive characteristics of the analyzed study sample at Waves I (1994–1996) and Wave V (2016–2018) (n = 7,069)

	Wave I	Wave V
Sex (women), n (%)	4,014 (56.8)	
Age, years	15.35 (1.34)	37.25 (1.53)
Race category		
White, n (%)	4,997 (71.6)	
Black, n (%)	1,412 (20.2)	
Native American, n (%)	115 (1.6)	
Asian, n (%)	456 (6.5)	
Parental income, $ thousand	1,045.14 (2985.88)	
Alcohol consumption		
Former drinker, n (%)		3,029 (42.8)
Guideline component met		
Physical activity, n (%)	2,228 (31.5)	1,534 (21.7)
Screen time, n (%)	2,859 (40.4)	5,913 (83.6)
Sleep duration, n (%)	4,063 (57.5)	4,093 (57.9)
None	1,219 (17.2)	433 (6.1)
All three guidelines	547 (7.7)	817 (11.6)
Depression CSD-5 item (0–15)		2.24 (2.43)
Medication for depression, n (%)		632 (8.9)
Depression, n (%)		1,481 (21.0)
Suicidal ideation, n (%)		332 (5.2)
Suicidal attempts		
Once, n (%)		44 (0.9)
Twice, n (%)		12 (0.3)
Three or four times, n (%)		9 (0.1)
Five or more times, n (%)		6 (0.1)

CSD, Center for Epidemiologic Studies Depression Scale

Table 2 shows the associations of physical activity, screen time, and sleep duration and all three guidelines during adolescence with depression and suicidal ideation in adulthood. Adolescents who met physical activity guideline had lower risk of depression at adulthood than those who did not meet any of these guidelines (IRR = 0.84, 95% CI 0.72 to 0.98). Also, those who met physical activity (IRR = 0.74, 95% CI 0.55 to 0.99) and all three guidelines (IRR = 0.59, 95% CI 0.32 to 0.98) had lower risk of suicidal ideation than adolescents who did not meet any of these guidelines.

**Table 2:** Incidence rate ratio for depression and suicidal ideation at Wave V associated with physical activity, screen time, and sleep duration guidelines and all the three during adolescence

	Depression		Suicidal ideation	
	IRR (95% CI)	p	IRR (95% CI)	p
Neither	1		1	
Physical activity only*	**0.84 (0.72 to 0.98)**	**0.029**	**0.74 (0.55 to 0.99)**	**0.049**
Screen time only*	1.04 (0.92 to 1.18)	0.565	0.91 (0.70 to 1.17)	0.459
Sleep duration only*	0.94 (0.83 to 1.06)	0.337	1.01 (0.78 to 1.29)	0.956
Meeting all three guidelines	0.92 (0.71 to 1.21)	0.573	**0.59 (0.32 to 0.98)**	**0.042**

IRR, incidence rate ratio.

Analysis adjusted by sex, race/ethnicity, parental income at Wave I, age at Wave V, depression score (5-item depression scale) at Wave V, and alcohol consumption at Wave V. * Mutual adjustments between physical activity, screen time, and sleep duration.

CI, confidence interval.

Trajectories of the 24-h movement guidelines from adolescence to adulthood are shown in Fig. 1. Overall, 26.8%, 89.7%, 60.5%, and 17.6% of the sample met physical activity, screen time, sleep duration, and all three guidelines in both waves, respectively. The percentage of participants who reported increased levels of physical activity, screen time, sleep duration, and all three guidelines from adolescence to adulthood was 19.4%, 79.5%, 54.4%, and 11.1%, respectively. In contrast, the percentage of the participants who reported decreased levels of physical activity, screen time, sleep duration, and all three guidelines from adolescence to adulthood was 80.6%, 20.5%, 45.6%, and 88.9%, respectively. Finally, 73.2%, 10.3%, 39.5%, and 82.4% of the participants did not meet the guidelines for physical activity, screen time, sleep duration, and all three guidelines in both moments, respectively.

Finally, Table 3 shows the associations of trajectories of physical activity, screen time, and sleep duration and all three guidelines from adolescence to adulthood with depression symptoms and suicidal ideation at Wave V. Individuals who met the guidelines for physical activity (IRR = 0.56, 95% CI 0.40 to 0.79), screen time (IRR = 0.87, 95% CI 0.72 to 0.98), and all three guidelines (IRR = 0.37, 95% CI 0.15 to 0.92) in both adolescence and adulthood had lower risk of depression symptoms than those who never met the guidelines. Also, those who did not meet physical activity (IRR = 0.91, 95% CI 0.77 to 0.99) and screen time guidelines (IRR = 0.83, 95% CI 0.72 to 0.98) in adolescence but did meet the guideline in adulthood had lower risk of depression symptoms than those who never met the guideline. Those who met the guidelines for screen time (IRR = 0.74, 95% CI 0.51 to 0.97) and all three guidelines (IRR = 0.12, 95% CI 0.06 to 0.33) in both adolescence and adulthood had lower risk of suicidal ideation than those who never met the guidelines. Additionally, individuals who did not meet all three guidelines in adolescence but did meet the guidelines in adulthood had lower risk of suicidal ideation than those who never met the guidelines (IRR = 0.81, 95% CI 0.45 to 0.89).

**Table 3:** Association between trajectories of physical activity, screen time, and sleep duration guidelines and all the three during between adolescence to adulthood with depression symptoms and suicidal ideation at Wave V

	Depression		Suicidal ideation	
	IRR (95% CI)	p	IRR (95% CI)	p
Physical activity guideline only				
Never met the guidelines	1		1	
Met at adolescence not at adulthood	1.02 (0.86 to 1.08)	0.129	0.74 (0.53 to 1.04)	0.086
Not met at adolescence but at adulthood	**0.91 (0.77 to 0.99)**	**0.047**	1.19 (0.82 to 1.73)	0.365
Met at adolescence and adulthood	**0.56 (0.40 to 0.79)**	**< 0.001**	0.81 (0.46 to 1.42)	0.469
Screen time guideline only				
Never met the guidelines	1		1	
Met at adolescence not at adulthood	0.95 (0.69 to 1.30)	0.740	1.18 (0.73 to 1.91)	0.508
Not met at adolescence but at adulthood	**0.83 (0.69 to 0.99)**	**0.039**	0.83 (0.60 to 1.17)	0.295
Met at adolescence and adulthood	**0.87 (0.72 to 0.98)**	**0.047**	**0.74 (0.51 to 0.97)**	0.024
Sleep duration guideline only				
Never met the guidelines	1		1	
Met at adolescence not at adulthood	0.94 (0.78 to 1.12)	0.479	0.93 (0.66 to 1.32)	0.700
Not met at adolescence but at adulthood	0.96 (0.79 to 1.16)	0.648	0.86 (0.58 to 1.27)	0.494
Met at adolescence and adulthood	0.90 (0.76 to 1.07)	0.256	0.97 (0.67 to 1.40)	0.869
Meeting all three guidelines				
Never met the guidelines	1		1	
Met at adolescence not at adulthood	1.12 (0.87 to 1.43)	0.374	0.61 (0.33 to 1.14)	0.306
Not met at adolescence but at adulthood	0.91 (0.72 to 1.14)	0.414	**0.81 (0.45 to 0.89)**	0.031
Met at adolescence and adulthood	**0.37 (0.15 to 0.92)**	0.032	**0.12 (0.06 to 0.33)**	0.002

IRR, Incidence rate ratio.

Analysis adjusted by sex, race/ethnicity, parental income at Wave I, age at Wave V, depression score (5-item depression scale) at Wave V, and alcohol consumption at Wave V.

* Interpret “meeting all three” results with caution given low frequency of individuals in “meets at adolescence and adulthood” category and suicidal ideation (1.2%).

## Discussion

To our knowledge, this study is the first to examine the association of trajectories in adherence to the 24-h movement guidelines from middle adolescence to adulthood with depression and suicidal ideation in adulthood. Overall, adherence to the 24-h movement guidelines in both adolescence and adulthood seems to be more strongly associated with lower risk of depression and suicidal ideation than never meeting the guidelines. Health practitioners should therefore encourage individuals to adhere to the 24-h movement guidelines from adolescence to adulthood to improve mental health.

### Depression and its relationship with 24-h movement guidelines

The evidence concerning physical activity and mental health effects is extensive and still growing [33]. One of the main findings of our study is that meeting with physical activity guideline at adolescence and adherence of these recommendations from adolescence to adulthood was associated with decreased risk of reporting future depression. Also, transitioning from nonadherence to adherence to physical activity guidelines was associated with lower risk of depression symptoms than those who never met the guideline. Our results corroborate the findings of a great deal of available evidence supporting the notion that physical activity can confer protection against the emergence of depression regardless of age and geographical region [34]. A recent meta-analysis of 15 prospective studies including more than 2 million person-years also shows that relatively small doses of physical activity were associated with substantially lower risks of depression [35]. Several biochemical and psychosocial factors are likely responsible for our finding. For instance, it is well known that individuals with depression have decreased levels of markers of neurogenesis and hippocampal volumes [36] and increased levels of inflammatory and oxidant markers [37] and that physical activity could attenuate these problems [34, 38]. For example, physical activity may increase hippocampal volume [39] and neurogenesis levels [40] and adjust the imbalance between anti- and proinflammatory and oxidant markers [41]. Participating regularly in physical activities has also shown to promote important psychological benefits, including the improvement of self-esteem, self-perception and identity, quality of life, confidence in ability, and self-efficacy [42].

For screen time, our results also suggest that individuals who met this guideline at both moments (i.e., adolescence and adulthood) had 13% lower risk of depression in comparison with individuals who never met this guideline. Also, those who did not meet screen time guideline in adolescence but did meet the guideline in adulthood had 17% lower risk of depression than those who never met the guideline. Again, two meta-analyses of prospective studies have corroborated that sedentary behavior is associated with an increased risk of depression in youths [43] and adults [44]. Additionally, a one-year prospective study also provided evidence of the link between screen time and depression among Canadian youths from the COMPASS study [23]. One hypothesis proposed to justify the association of sedentary time and depression risk is that this behavior may displace physical activity [45], which, as mentioned above, seems to be beneficial in reducing the risk of depression [34, 38]. Another hypothesis focused on social/psychological theories is that increased sedentary time may prevent individuals from social interactions and thereby increase their risk for depression [46]. However, future research is needed to further investigate potential neurobiological and psychosocial mechanisms of the effect of sedentary behavior on depression.

Emerging evidence has established the importance of taking an integrative, rather than isolated, approach to examining the influence of 24-h movement guidelines on the risk of depression [20, 21]. Our study suggested that maintaining all the 24-h movement guidelines from adolescence to adulthood seems to be associated with 63% lower risk of depression at follow-up. This finding is consistent with that of Sampasa-Kanyinga et al. [21], who conducted a systematic review and found that adherence to the 24-h movement guidelines among children and adolescents is associated with better mental health status. Among adults, associations between meeting the 24-h movement guidelines and depression vary across studies. However, all these studies had a cross-sectional design and small sample sizes. For example, our finding is contrary to previous studies that have suggested that meeting all three 24-h movement guidelines was not associated with depression and other mental health outcomes (e.g., anxiety, stress) [47, 48]. A possible explanation for these results might be that the abovementioned studies used compositional data analysis, an approach that enables studying the combined effects of different movement-related behaviors [14].

### Suicidal ideation and its relationship with 24-h movement guidelines

Evidence is accumulating on the mental health effects of modifiable lifestyle behaviors among adolescents and adults [49, 50]. Consistent with the literature, our research found that individuals who maintained adherence to screen time guidelines from adolescence to adulthood showed 26% decreased risk of suicidal ideation. These results reflect those of Coyne et al. [49], who also found that a high level of social media, television use or video games in early adolescence (followed by a marked increase over time) was most predictive of suicide risk in emerging adulthood, especially for girls. As suggested by Lin [50], viewing television and videos and playing video games are often (though not always) a solitary activity and therefore may displace face-to-face interactions with others, which may in turn decrease feelings of belongingness. Therefore, it appears that meeting with the screen time guidelines over the course of life may be protective against developing suicide risk over time.

Since single health behaviors, as unhealthy behaviors, tend to cluster, it seems to be important to analyze them jointly rather than individually [12, 13]. Thus, findings from the SEYLE study among 12,395 adolescents from eleven European countries [51] defined for the first time a group named “invisible-risk”, which includes adolescents who have reduced sleep, low physical activity and high media use. These groups of adolescents showed high rates of depression, anxiety, and suicidal ideation [51]. Furthermore, this finding is consistent with that of Sampasa-Kanyinga et al. [22], who suggested that adherence to the 24-h movement guidelines among adolescents was related to lower risk of suicidal ideation in older boys. Similarly, our results showed that those who met all three guidelines during adolescence and those individuals who maintained the 24-h movement guidelines from adolescence to adulthood had lower risk of suicidal ideation. Additionally, transitioning from nonadherence to adherence to 24-h movement guidelines was associated with 19% lower risk of suicidal ideation in adulthood compared to those who never met the guidelines. Although the causality of the relationships between these risk behaviors and suicide thoughts remains unclear, this psychiatric disorder is already known to often show bidirectional relationships with reduced sleep [52], low levels of physical activity [9], and high screen time use among adults [49]. Whereas attention often focuses on substance abuse (e.g., alcohol, tobacco, drugs) among U.S. adults [53], the risk behaviors identified here need to be considered, and special attention should be given to encouraging adequate sleep duration, participation in regular physical activity, and using screen time moderately across life.

There are a few important limitations to the current study. First, longitudinal data were self-reported, which are subject to many sources of error and bias, including reporter bias or recall bias. For instance, the total amount of physical activity could have been overestimated by social approval or social desirability [54] and self-reported depression and suicidality tend to be underestimated [55]. Moreover, it is important to highlight that movement behaviors disturbances and depression have a bidirectional association; depression also influences sleep [56] and physical activity levels [57]. Second, diagnostic interviews would allow a better understanding of the relationship between 24-h movement behaviors and depression and suicidal ideation. Also, since depression was determined with self-reported questions and/or prescription medication-use for depression in the previous 4 weeks, we were unable to classify it as clinical depression or mild depressive symptoms. Third, we classified respondents as meeting physical activity guidelines when they performed MVPA five or more times per week and not as the accumulation of at least 60 min per day of MVPA; thus, the results need to be interpreted with caution. Also, the physical activity instrument used is limited in scope since does not include a school component and give no indication of time, which makes it really difficult to pinpoint if youth met the 60 min of MVPA per day recommendation. Fourth, dataset from 1994 to 1996 is being used to gauge meeting a guideline made later. Fifth, sedentary behaviors use has changed in the last two decades (e.g., from traditional television programming to streaming), what has caused an increase in screen time use. Future studies are needed to examine whether some screen time behaviors, such as engaging in healthy educational computers or television programs, have less harmful or protective effects on mental health than other screen time behaviors. Sixth, there was no information on pharmacotherapy or the possibility of access to treatment for mental disorders. Finally, other important covariates such as childhood adverse experiences and access to safe environments to be physically active have potential to change the observed relationship but were not included in our analysis. Regardless of these limitations, the major advantage of our study is the inclusion of a large and representative sample of U.S. individuals followed up for 22 years.

## Conclusion

Meeting the 24-h movement guidelines from adolescence to adulthood seems to be associated with lower risk of depression and suicidal ideation. Given the large burden of mental health [1] and low adherence to the 24-h movement guidelines worldwide [17], supporting individuals to remain adherent to these guidelines from adolescence to adulthood appears to be a worthwhile public health investment, with potential benefits for multiple aspects of physical, mental, and social health. Multidisciplinary interventions should be implemented to raise public awareness of the adverse consequences of insufficient physical activity and sleeping time and excessive screen time, and its associations with mental disorders such as depression and suicidal ideation via school and family strategies. In addition to the current public health messages focused on drug abuse and delinquency, a key policy priority should be guaranteeing appropriate systems, services and support for the risk behaviors identified here, with special attention to encouraging sufficient sleep and physical activity, and using screen time moderately throughout life.

## Electronic supplementary material

Below is the link to the electronic supplementary material.

Supplementary Material 1: STROBE Statement- checklist of items that should be included in reports of observational studies

## Data Availability

Data sharing is not applicable to this article as no datasets were generated or analysed during the current study.

## Abbreviations

CES-D: Center for Epidemiologic Studies Depression Scale.
IRR: incidence rate ratio.
MVPA: moderate-to-vigorous physical activity.
U.S.: United States.
